# Algorithm vs clinical experience: controlled ovarian stimulations with follitropin delta and individualised doses of follitropin alpha/beta

**DOI:** 10.1530/RAF-23-0045

**Published:** 2024-02-28

**Authors:** Irene Gazzo, Francesca Bovis, Denise Colia, Fausta Sozzi, Mauro Costa, Paola Anserini, Claudia Massarotti

**Affiliations:** 1 Department of Neurosciences, Rehabilitation, Ophthalmology, Genetics and Maternal and Child Health (DINOGMI Department), University of Genoa, Genova, Italy; 2 IRCCS Ospedale Policlinico San Martino, Genova, Italy; 3 Department of Health Sciences, University of Genoa, Genova, Italy; 4 Reproductive Medicine Unit, Ospedale Evangelico Internazionale, Genova, Italy; 5 Physiopathology of Human Reproduction Unit, IRCCS Ospedale Policlinico San Martino, Genova, Italy

**Keywords:** follitropin delta, controlled ovarian stimulation, OHSS, ART

## Abstract

In the registration trials, follitropin delta was compared with a fixed dose of 150 UI of follitropin alpha/beta, finding higher chances to reach a target response of 8–14 oocytes compared to controls. For this reason, follitropin delta is marketed as particularly useful in expected hyper-responder patients. The main outcome of this study is to report if comparable results are reached in a real-life scenario with follitropin alpha/beta doses chosen by the physician, based on patients’ characteristics. This is a retrospective study performed in two public fertility centres. All first cycles from January 2020 to June 2022 with either follitropin delta (cases) or alpha/beta (controls) in patients with anti-Müllerian hormone >2.5 ng/mL were compared by an inverse probability weighting approach based on propensity score. The follitropin total dose was higher in controls (1179.06 ± 344.93 vs 1668.67 ± 555.22 IU, *P* < 0.001). The target response of 8–14 oocytes was reached by 40.2% of cases and 40.7% of controls (odds ratio (OR): 0.99, 95% confidence interval (CI): 0.65–1.53, *P* = 0.98). Fewer than 8 oocytes were collected in 24.1% of cases and 22% of controls (OR: 1.10, 95% CI: 0.71–1.69, *P* = 0.67); more than 14 oocytes in 35.7% of cases and 37.3% of controls (OR: 0.83, 95% CI: 0.54–1.28, *P* = 0.40). Our experience did not find worse results in term of proportion of patients who reached the target response with an algorithm-chosen dose of follitropin delta compared to a personalised starting dose of follitropin alpha/beta, with follitropin delta having the advantage of objectivity. However, larger numbers are needed to confirm these results.

**Lay summary:**

The starting dose of the drugs used to stimulate the ovaries in IVF (gonadotropins) is usually decided by the doctor, using their clinical experience and expertise and tailored to the individual patient. Recently one type of stimulating drug (follitropin delta) was marketed with an algorithm for deciding the starting dose based on the patient’s anti-Müllerian hormone (AMH) levels and weight. In the initial trials, it was compared with a fixed dose of standard follitropins (alpha/beta), and it was found to reduce the likelihood of an excessive response in patients at risk of ovarian hyperstimulation syndrome. We report on these results, in terms of number of eggs obtained, in patients with an expected high ovarian response, compared to doses of standard follitropins that were not fixed, but personalised, to see if this did not make a difference. We found similar results in the two groups, suggesting that using the algorithm to decide the dose of follitropin delta does not work less well than a personalised starting dose of follitropin alpha/beta, but has the advantage of being objective.

## Introduction

The development of algorithms and machine learning models for reproductive medicine is steadily growing in recent years (Wang *et al.* 2019). Among them, several potential models for calculating a personalised starting dose of gonadotropins are published in literature (Li *et al.* 2021, Marino *et al.* 2022), but no one is routinely used in fertility units worldwide, except for the follitropin delta algorithm.

Follitropin delta is a recombinant FSH (r-FSH) produced in a human-derived cell line PER.C6, with has a different glycosylation profile and therefore lower clearance than traditional r-FSH preparations (follitropin alpha and beta, produced in Chinese Hamster Ovary cells). Due to these differences, follitropin delta induces a higher ovarian response in humans than existing r-FSH preparations when administered at equal doses of biological activity (Olsson *et al.* 2014). To facilitate clinical use, it was marketed to be used in controlled ovarian stimulation (COS) in a fixed daily dose determined by an algorithm based on patients’ anti-Müllerian hormone (AMH) and weight (Nyboe Andersen *et al.* 2017). The algorithm was defined by manufacturers based on the results of the phase two registrational trial, where different dosages were compared, and through pharmacokinetic and pharmacodynamic simulation (Arce *et al.* 2014). The phase three registration randomised blinded trial then demonstrated follitropin delta is non-inferior to conventional ovarian stimulation in terms of implantation rates and ongoing pregnancy rates and underlined that more women reached the target response of 8−14 oocytes compared to controls (Nyboe Andersen *et al.* 2017). Multiple real-life case series reported optimal efficacy in high responder patients (Blockeel *et al.* 2022), with a low risk of ovarian hyperstimulation syndrome (OHSS) (Ishihara *et al.* 2021, Yacoub *et al.* 2021).

The main criticism to the available evidence is that all trials compare follitropin delta to a standard dose of 150 IU of follitropin alpha/beta, and not to a personalised dose, as it would be more representative of actual clinical practice (Montenegro Gouveia *et al.* 2022). In most fertility units, in a real-life setting, the gonadotropin dose for the first COS cycle is decided by the clinician taking into account the patient’s age, serum AMH levels and antral follicle count and, to a lesser extent, other parameters such as early follicular phase FSH levels and patient’s weight (Keck *et al.* 2005, Sighinolfi *et al.* 2017, Leijdekkers *et al.* 2019). Moreover, the result is influenced by the clinician’s experience and expertise, since there is no recommended standard decision process around the world (Farquhar & Marjoribanks 2018). In patients expected to be hyper-responders, the balance between obtaining a number of oocytes to optimise the chances of live birth but not too many for the risk of OHSS is particularly delicate (Briggs *et al.* 2015).

To fill the gap between registration trials and real-life experiences of fertility units, in this retrospective multicentric study we compare for the first time the ovarian response to COS with an individualised dose of follitropin alpha/beta versus the algorithm-based dose of follitropin delta in patients expected to be hyper-responders.

## Materials and methods

### Study design, size, duration

This is a multicentric retrospective study, performed by two public fertility centres in Genoa, Italy. Follitropin delta was introduced in the Italian market in 2019, with a strong emphasis on its potential in reaching a target response in expected hyper-responder patients. We retrospectively revised our databases from January 2020 to June 2022 to collect all first cycles with follitropin delta in women with predicted hyper response. All first cycles with follitropin alpha/beta in women with comparable characteristics in the same time period served as controls. We excluded repeated cycles since the dose choice would have been influenced by the previous one(s) in both cases and controls.

Inclusion criteria were age between 18 and 43 years; AMH ≥2.5 ng/mL; first ovarian stimulation cycle for in vitro fertilisation with either follitropin delta or follitropin alpha/beta. Exclusion criteria were BMI below 18 or over 30 kg/m^2^, absence or denial of consent for the use of anonymized data for clinical research and publication.

### COS procedures

Ovarian stimulation was started on day 2 of the menstrual cycle, after hormonal pretreatment (combined hormonal contraception). On the same day, follicular antral count and patient’s weight were collected. All other demographic and clinical parameters, including AMH levels, were available in patients’ charts. Serum AMH was measured by Roche Elecsys AMH system, range: 0.01–23 ng/mL, repeatability: 1.0–1.6%, CV: 0.055–19.0 ng/mL (Elecsys, Roche Diagnostics).

If the stimulation was performed with follitropin delta, the starting dose was calculated using the dedicated algorithm. Follitropin alpha or beta starting doses were defined based on physician choice, evaluating parameters such as AMH serum level, patient’s age, patient’s weight and antral follicular count, all in light of clinical experience. Based on these parameters, the standard starting dose of 150 UI was increased up to a maximum of 225 UI or decreased down to a minimum of 100 UI. No dose higher than 225 UI was used because the selected patients had a good ovarian reserve and a good ovarian response was expected. Once the dose was defined, no dose adjustment was performed during the stimulation. The same two expert physicians (PA and MC) supervised all the cycles. For data analysis we considered 10 μg of follitropin delta as comparable to 150 UI of follitropin alpha/beta (Arce *et al.* 2020), for better comparability between groups.

GnRH antagonist (Ganirelix 0.25 mg) was administered after 5 to 7 days of stimulation, based on oestrogen levels (>200 pg/mL) and/or ultrasonographic number and dimension of ovarian follicles (at least 3 follicles >11 mm or one leading follicle ≥13 mm). When the lead follicle(s) reached 18 mm size, highly purified human chorionic gonadotropin 10000 IU or GnRH agonist 0.2 mg were used to induce final oocytes maturation. Oocytes retrieval was performed 35–36 h later.

### Outcomes

The primary outcome of this study is to compare the proportion of patients who reached a target response of 8–14 oocytes in the two treatment groups (follitropin delta vs follitropin alpha/beta). A number of oocytes between 8 and 14 was selected as target response for better comparability with the follitropin delta registration studies (Nyboe Andersen *et al.* 2017) and with the existing literature that defines it as the optimal balance between the chances of clinical pregnancy and the risk of OHSS (Drakopoulos *et al.* 2016, Bachmann *et al.* 2022). Patients whose cycle was stopped due to an inadequate response were included in the analyses, their oocyte count was recorded as zero.

Secondary outcomes included number of cycles stopped before eggs retrieval, COS duration, follitropin total dose, cycles with <8 or >14 oocytes retrieved and number of mature metaphase II (MII) oocytes retrieved.

### Data analysis

Baseline patients’ characteristics were described as proportions (percentages) for categorical variables, means and standard deviation (s.d.) for continuous variables. Owing to the presence of some missing at random values, to make efficient use of the available data, we used multiple imputation of missing values for missing data. Imputation was performed using chained equations (Burgess *et al.* 2013), where each incomplete variable is imputed by a separate model and implemented through the Multiple Imputation by Chained Equation (MICE) algorithm (‘mice’ R package).

Baseline disease and demographic characteristics were summarised by group and overall, using descriptive statistics, and were compared between treatment groups using the standardised mean difference (s.m.d.) as calculated according to Cohen’s *d* effect size. A Cohen’s *d* effect size >0.1 denotes meaningful imbalance in the baseline covariates (Jacob 1988). Using s.m.d. (that is the mean difference expressed in units of s.d.) allows for a meaningful and standardized assessment of the magnitude of differences between groups, especially when dealing with outcomes measured on different scales or with varied units.

In order to address the baseline disparities between treatment groups, an inverse probability weighting (IPW) approach based on propensity score (PS) was employed. The weights correspond to the inverse of the conditional PS of receiving the follitropin delta treatment. The PS for each patient was calculated as a probability from a logistic regression model that had treatment as the dependent variable (follitropin delta vs follitropin alpha/beta) and the following baseline variables as independent covariates: age, fertility unit, BMI, AMH, presence of severe male infertility, presence of PCOS. We used stabilised trimmed weights (Austin & Stuart 2015) (any weights exceeding a predefined threshold were each set to that threshold) to mitigate the impact of extremely higher or lower weights on the variability of the estimated treatment effect. The threshold (1%) was based on the quantiles of the distribution of the weights.

We employed an IPW logistic regression model to evaluate variations in treatment outcomes regarding the target response of 8–14 oocytes, cycles with fewer than 8 or more than 14 oocytes retrieved, and freeze-all cycles. We utilized an IPW Poisson regression model to examine differences in the number of MII oocytes retrieved, and an IPW linear regression model was applied to assess variations in COS duration and the total dose of follitropins between treatments. The application of different regression models was driven by the distinct nature of the study outcomes: we employed IPW logistic regression for binary outcomes, IPW Poisson regression models for count variables, and IPW linear regression when dealing with continuous outcomes. A *P*-value <0.05 was considered significant. SAS 9.4 (SAS Institute Inc., Cary, NC, USA) and R (v 4.1.3) were used for the computation.

## Results

After the retrospective database analysis, 483 cycles (121 with follitropin delta and 362 with follitropin alpha/beta) fitted the inclusion/exclusion criteria and were selected for this study.

Missing data ranged from 0.2% to 11.4% and were attributed to missing data in the centres’ documentation. To address missing data, we employed a multiple imputation technique, as detailed in the ‘Materials and methods’ section.

The unweighted characteristics of the patients included in the analysis, according to the treatment groups, were reported in Supplementary Table 1 (see section on supplementary materials given at the end of this article). Patients treated with follitropin delta are generally younger, with a higher BMI and lower AMH levels. The weighted characteristics were well balanced between the treatment groups with a residual imbalance (s.m.d. = 0.11) persisted for the AMH level (Table 1).

**Table 1:** Inverse probability-weighted demographic and clinical characteristics. Data are presented as *n* (%) or as mean ± s.d.

	Follitropin α/β	Follitropin δ	*P*	s.m.d.*
*n*	362	121		
Fertility centre				
1	110 (30.4)	40 (33.1)	0.76	0.032
2	252 (69.6)	81 (66.9)		
Age, years	34.33 ± 4.44	34.22 ± 4.05	0.80	0.026
BMI, kg/m^2^	22.33 ± 3.29	22.36 ± 3.60	0.95	0.007
AMH, ng/mL	5.87 ± 4.59	5.46 ± 2.82	0.25	0.108
PCOS	227 (62.7)	73 (60.33)	0.72	0.039
Severe male factor	147 (40.6)	47 (38.8)	0.85	0.021

*Cohen’s *d* values (effect sizes) represent standardised mean or proportion differences. Absolute values of *d* > 0.10 were considered clinically meaningful.

AMH, anti-Müllerian hormone; BMI, body mass index; PCOS, polycystic ovary syndrome, s.m.d., standardised mean difference.

The IPW-adjusted treatment effect estimates and their corresponding 95% confidence intervals (CI) were reported in Table 2. The results of both univariable and multivariable analyses are available in Supplementary Table 2.

**Table 2:** Clinical outcome of cycles with follitropin δ vs α/β after IPW adjustment.

	IPW-adjusted analysis (*n* = 483)	
	OR (95% CI)	*P*
Target response (8–14 oocytes)^ǂ^	0.99 (0.65–1.53)	0.98
Less than 8 oocytes^ǂ^	1.10 (0.71–1.69)	0.67
More than 14 oocytes^ǂ^	0.83 (0.54–1.28)	0.34
Freeze-all cycles^ǂ^	1.18 (0.78–1.79)	0.43
MII oocytes^ǂǂ^	0.95 (0.88–1.02)^†^	0.17
COS duration^§^	0.21 (−0.24 to 0.66)*	0.36
Total dose^§,++^	−497.16 (−621.57 to –372.75)*	<.0001

^ǂ^Patients were compared between arms using a regression logistic model; ^ǂǂ^Estimates and *P*-values were calculated with the use of a Poisson regression model; ^§^Estimates and *P*-values were calculated with the use of a regression linear model; ^++^ *n* = 217; ^†^Value is RR (95% CI); *Value is *β* coefficient (95% CI).

CI, confidence interval; COS, controlled ovarian stimulation: MII, metaphase II; OR, odds ratio; RR, rate ratio.

### Primary outcome

Compared to the follitropin alpha/beta-treated group, the proportion of patients who reaching the target response (8–14 oocytes) in the follitropin delta-treated group was not statistically different (odds ratio (OR) = 0.99; 95% CI: 0.65–1.53; *P* = 0.98). The absolute probability of reaching the target response for the follitropin delta-treated patients was 37% (95% CI: 29–46%), while for the follitropin alpha/beta-treated group was 37% (95% CI: 32–42%).

### Secondary outcomes

We found no evidence of difference between follitropin alpha/beta and follitropin delta treatment in the proportion of patients with less than eight oocytes (OR = 1.10; 95% CI: 0.71–1.69; *P* = 0.67) or with more than 14 oocytes (OR = 0.83; 95% CI: 0.54–1.28; *P* = 0.34) or with freeze-all cycles (OR = 1.18; 95% CI: 0.78–1.79; *P* = 0.434). We found no conclusive evidence of difference between groups in the number of MII oocytes (rate ratio (RR) = 0.95; 95% CI: 0.88–1.02; *P* = 0.17). The results in COS duration did not reach statistical significance (*β* coefficient = 0.21; 95% CI: –0.24 to 0.66; *P* = 0.36).

The only statistical difference between follitropin delta and follitropin alpha/beta was observed in the total dose administered (*β* coefficient = −497.16; 95% CI: −621.57 to −372.75; *P* < 0.0001). This analysis was conducted on a subset of 217 patients (44.9%), because data for the remaining patients was unavailable.

## Discussion

We report for the first time a real-life example of follitropin delta usage compared to an individualised dose of follitropin alpha/beta, chosen by expert physicians.

The definition of ‘successful ovarian stimulation’ is challenging. Our final aim is and must always be the live birth of a healthy child, but in the last decades, as reproductive technologies became less and less experimental, there has been a necessary shift in endpoints, from ‘pregnancy’ to ‘safe pregnancy’ (Bortoletto & Romanski 2021). With this aim to guide the physician, it emerged the necessity of reducing iatrogenic harm at every stage of the process, without reducing the chances of success. OHSS, defined ‘the great enemy’ of the reproductive physician, is now seen as evitable thanks to strategies such as the GnRH agonist trigger and cycles’ segmentation (Mourad *et al.* 2017), but these strategies do not completely eliminate the chance of severe symptoms requiring hospitalisation (Hajizadeh *et al.* 2023). So, while there is still debate on the optimal number of oocytes to retrieve for optimal chances of pregnancy (Drakopoulos *et al.* 2016, Bachmann *et al.* 2022), it makes sense to aim to collect a good number of oocytes but not too many.

In expected hyper-responders there are mainly two challenges when performing COS: to avoid a suboptimal response or the selection of a dominant follicle, and to avoid an excessive response.

As for the reaching of a target ovarian response (defined, for comparability with the registration trial, as 8−14 oocytes), a similar percentage reached the outcome in the two groups. Significantly, those who did not, were similarly distributed among insufficient and excessive responses, demonstrating once again the comparability of the two methods of dose-choosing. In favour of follitropin delta we can mention the independence from the physician’s expertise and the use of a minor dose to reach similar outcomes. The high numbers of segmented cycles (approximately half of the cycles in the two groups) is to be expected in such a cohort, and once again the follitropin used was not influential on the results.

The main limitation of this study lies in its retrospective nature. It is subject to the common limitations associated with non-randomised comparisons. To address the potential bias resulting from the absence of randomisation, we employed IPW analysis. Cases and controls were different women and we know that the ovarian response to COS is largely subjective: the IPW adjustment was useful also in reducing this bias, making the two groups comparable regarding all the major characteristics involved in ovarian response. The decision of a personalised dose of follitropin alpha/beta will always be physician dependent, but all cases were supervised by the same two expert physicians for uniformity. Moreover, there were no significant differences among results in the two clinics.

In conclusion, the results of not inferiority of follitropin delta compared to a personalised dose of follitropin alpha/beta must be corroborated by larger and/or randomised studies, as our relatively small sample size cannot guarantee definitive answers. However, our experience reports a snapshot clinical reality and did not find a difference in results between an algorithm-chosen dose of follitropin delta and a personalised starting dose of follitropin alpha/beta based on clinical practice, with the first having the advantage of objectivity.

## Supplementary Materials

Supplementary Tables

## Declaration of interest

Claudia Massarotti is an associate editor of *Reproduction and Fertility*. Claudia Massarotti was not involved in the review or editorial process for this paper, on which she is listed as an author. The authors report no conflict of interest related to the present paper.

## Funding

This research did not receive any specific grant from funding agencies in the public, commercial, or not-for-profit sectors.

## Author contribution statement

PA, MC and CM designed the study. I.G. wrote the first draft of the manuscript, CM, PA and MC revised it for important intellectual content. IG, FS and DC collected patients’ data and treated them for COS cycles. FB performed the statistical analysis. PA and MC coordinated the group. All authors contributed to critical discussion. All authors revised intermediate versions of the manuscript, suggested improvements, and read and approved the final version of the manuscript.
