# Supplementary material for: Algorithm vs clinical experience: controlled ovarian stimulations with follitropin delta and individualised doses of follitropin alpha/beta
Source: Reprod Fertil. 2024 Feb 28;5(1):e230045. doi: 10.1530/RAF-23-0045 (PMC10959055; doi:10.1530/RAF-23-0045)
Supplement: Supplementary Tables [file supplementary_tables.pdf]

Supplementary tables

Supplementary table 1 – Unweighted baseline characteristics of the patients included in the analysis

	Overall	Follitropin alpha/beta	Follitropin delta	p	SMD*
	483	362	121		
Fertility Center, n(%)				0.003	0.317
**1**	146 (30.2)	96 (26.5)	50 (41.3)		
**2**	337 (69.8)	266 (73.5)	71 (58.7)		
**Age**, years (mean (SD))	34.35 (4.35)	34.61 (4.38)	33.57 (4.17)	0.002	0.243
**BMI**, kg/m^2(mean (SD))	22.34 (3.37)	22.23 (3.23)	22.66 (3.75)	0.221	0.124
**AMH**, ng/ml (mean (SD))	5.90 (4.47)	6.08 (4.91)	5.37 (2.68)	0.131	0.179
**PCOS**, n(%)	304 (62.9)	229 (63.3)	75 (62.0)	0.886	0.026
**Severe male factor**, n(%)	196 (40.6)	150 (41.4)	46 (38.0)	0.578	0.070

* Cohen's d values (effect sizes) represent standardised mean or proportion differences. Absolute values of d>0.10 were considered clinically meaningful.

SD: standard deviation, BMI= body mass index; AMH = antiMullerian hormone; PCOS= polycystic ovary syndrome, SMD= standardised mean difference

Supplementary table 2 - Clinical outcome of cycles with follitropin delta vs alpha/beta: univariable and multivariable analysis and inverse probability weighting (IPW) adjusted analysis

	**Univariable analysis N=483**		**Multivariable analysis* N=483**		**IPW-adjusted analysis N=483**	
	**OR (95%CI)**	**p-value**	**OR (95%CI)**	**p-value**	**OR (95%CI)**	**p-value**
**Target response (8-14 oocytes)** ‡	1.02 (0.67-1.56)	0.929	1.02 (0.66-1.58)	0.912	0.99 (0.65-1.53)	0.975
**Less than 8 oocytes** ‡	1.01 (0.65-1.55)	0.971	1.14 (0.72-1.80)	0.575	1.10 (0.71-1.69)	0.669
**More than 14 oocytes** ‡	0.89 (0.58-1.37)	0.610	0.76 (0.48-1.20)	0.240	0.83 (0.54-1.28)	0.399
**Freeze-all cycles**‡	1.02 (0.67-1.54)	0.935	1.21 (0.78-1.86)	0.396	1.18 (0.78-1.79)	0.434
	**RR (95%CI)**	**p-value**	**RR (95%CI)**	**p-value**	**RR (95%CI)**	**p-value**
**MII oocytes** ‡‡	1.00 (0.95-1.08)	0.906	0.94 (0.87-1.01)	0.081	0.95 (0.88-1.02)	0.168
	**Beta coefficient (95%CI)**	**p-value**	**Beta coefficient (95%CI)**	**p-value**	**Beta coefficient (95%CI)**	**p-value**
**COS duration** §	0.27 (from -	0.244	0.25 (from -	0.279	0.21 (from -	0.356

	0.18 to 0.71)		0.20 to 0.69)		0.24 to 0.66)	
**Total dose** [§,++]	-485.37 (from -609.89 to -360.86)	<.0001	-467.83 (from -612.01 to -323.65)	<.0001	-497.16 (from -621.57 to -372.75)	<.0001

*center, age, BMI, amh, PCOS and male infertility as covariates

ǂ Patients with CCOC were compared between arms using a regression logistic model

[#]Estimates and p-values were calculated with the use of a poisson regression model

[§]Estimates and p-values were calculated with the use of a regression linear model

[++] N=217

MII= metaphase II; COS= controlled ovarian stimulation: OR = odds ration; RR= rate ratio; CI= confidence interval
